# White matter hyperintensities are common in midlife and already associated with cognitive decline

**DOI:** 10.1093/braincomms/fcz041

**Published:** 2019-12-09

**Authors:** Tracy d’Arbeloff, Maxwell L Elliott, Annchen R Knodt, Tracy R Melzer, Ross Keenan, David Ireland, Sandhya Ramrakha, Richie Poulton, Tim Anderson, Avshalom Caspi, Terrie E Moffitt, Ahmad R Hariri

**Affiliations:** 1 Department of Psychology & Neuroscience, Duke University, Durham, NC 27708, USA; 2 New Zealand Brain Research Institute, 66 Stewart Street, Christchurch 8011, New Zealand; 3 Department of Medicine, University of Otago, 2 Riccarton Avenue, Christchurch 8011, New Zealand; 4 Christchurch Radiology Group, 6/242 Ferry Road, Waltham, Christchurch 8011, New Zealand; 5 Dunedin Multidisciplinary Health and Development Research Unit, Department of Psychology, University of Otago, Dunedin 9016, New Zealand; 6 Social, Genetic, & Developmental Psychiatry Research Centre, Institute of Psychiatry, Psychology, & Neuroscience, King’s College London, London SE5 8AF, UK; 7 Department of Psychiatry and Behavioral Sciences, Duke University School of Medicine, Durham, NC 27708, USA; 8 Center for Genomic and Computational Biology, Duke University, Durham, NC 27708, USA

**Keywords:** white matter hyperintensity, cognition, cognitive decline, dementia risk

## Abstract

White matter hyperintensities proliferate as the brain ages and are associated with increased risk for cognitive decline as well as Alzheimer’s disease and related dementias. As such, white matter hyperintensities have been targeted as a surrogate biomarker in intervention trials with older adults. However, it is unclear at what stage of aging white matter hyperintensities begin to relate to cognition and if they may be a viable target for early prevention. In the Dunedin Study, a population-representative cohort followed since birth, we measured white matter hyperintensities in 843 45-year-old participants using T_2_-weighted magnetic resonance imaging and we assessed cognitive decline from childhood to midlife. We found that white matter hyperintensities were common at age 45 and that white matter hyperintensity volume was modestly associated with both lower childhood (*ß* = −0.08, *P *=* *0.013) and adult IQ (*ß*=−0.15, *P *<* *0.001). Moreover, white matter hyperintensity volume was associated with greater cognitive decline from childhood to midlife (*ß*=−0.09, *P *<* *0.001). Our results demonstrate that a link between white matter hyperintensities and early signs of cognitive decline is detectable decades before clinical symptoms of dementia emerge. Thus, white matter hyperintensities may be a useful surrogate biomarker for identifying individuals in midlife at risk for future accelerated cognitive decline and selecting participants for dementia prevention trials.

## Teaser

White matter hyperintensities, which are associated with dementia risk in older adults, were found to be common in a population-representative birth cohort at age 45 and already associated with cognitive decline since childhood. This suggests they may be useful as surrogate biomarkers in dementia prevention trials targeting midlife.

## Graphical Abstract

**Graphical Abstract:**
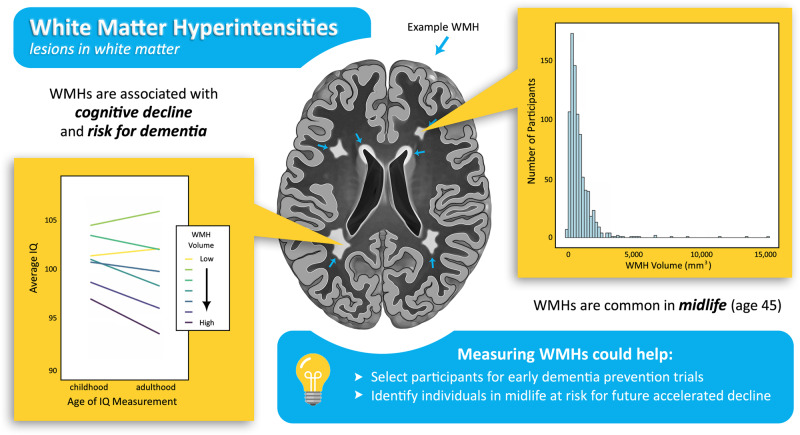

## Introduction

To address rising economic and health burdens due to Alzheimer’s disease and related dementias (ADRD), government funding for aging research has more than quadrupled in recent years (Kaiser, 2018). However, the success of this investment hinges on developing surrogate biomarkers—biological measures that are part of the putative disease pathway and are measurable before the onset of clinical symptoms—so that prevention can target at-risk individuals before cerebral decline has taken hold. Successful surrogate biomarkers would allow clinicians to assess risk, monitor sub-clinical disease progression and intervene before clinically significant dementia symptoms manifest.

Research shows that white matter hyperintensities (WMHs) are one such surrogate biomarker of cognitive decline and ADRD that can be measured in the brains of older adults (Cees De Groot *et al.*, 2000; Lee *et al.*, 2016). As the brain ages, it begins to accrue small microbleeds and lesions in white matter that are detectable as WMHs using fluid-attenuated inversion recovery (FLAIR) magnetic resonance imaging (MRI) (Iadecola, 2013). While WMHs are uncommon in adults before age 30 (Habes *et al.*, 2016), they are detectable in <90% of individuals by age 65 (Longstreth *et al.*, 1996). In older adults, WMHs are associated with multiple dementia risk factors, including increasing age, hypertension, stroke, brain atrophy and cognitive ability (Prins and Scheltens, 2015). Longitudinal studies in older adults have reported that the spread of WMHs contributes to elevated risk for ADRD and coincides with age-related cognitive decline (Debette and Markus, 2010). Furthermore, baseline WMH load at mean age 62 can predict the onset of dementia up to 20 years later (Coker *et al.*, 2019).

Consequently, WMHs have been targeted as a surrogate biomarker for dementia prevention trials (Debette and Markus, 2010). However, these trials have produced mixed results (Prins and Scheltens, 2015). A limitation of existing trials is that they have targeted older adults in their 60s, 70s and 80s. Older brains are characterized by age-related deterioration and may be less responsive to intervention (Sperling *et al.*, 2014; Moffitt *et al.*, 2017). One solution is to assess WMHs in midlife, a time when the brain may be more responsive to interventions and has yet to be affected by decades of age-related organ decline. It is known that WMHs predict cognitive decline and risk for ADRD in older adults (Valdés Hernández *et al.*, 2013), but it is not known when WMHs accumulate sufficiently to be associated with early cognitive decline.

Here, we tested the hypotheses that WMHs are detectable in midlife and already associated with cognitive decline from childhood in a population-representative birth cohort aged 45 years. Support for these hypotheses would provide novel evidence that WMHs could be a surrogate biomarker of risk in the general population as early as midlife, allowing for earlier—and potentially more effective—interventions for cognitive decline and ADRD.

## Materials and methods

### Study design and population

Participants were members of the Dunedin Multidisciplinary Health and Development Study, a longitudinal investigation of health and behaviour in a population representative birth cohort. The full cohort (*N* = 1037; 91% of eligible births; 52% male) comprises all individuals born between April 1972 and March 1973 in Dunedin, New Zealand, who were eligible based on residence in the province and who participated in the first assessment at age 3 years. The cohort represents the full range of socioeconomic status in the general population of South Island, New Zealand (Poulton *et al.*, 2015). The cohort matches the New Zealand National Health and Nutrition Survey on adult health indicators (e.g. body mass index, smoking, primary-care visits) and the NZ census on educational attainment. The cohort is primarily white (93%), which matches the demographics of the South Island (Poulton *et al.*, 2015). Assessments were carried out at birth and at ages 3, 5, 7, 9, 11, 13, 15, 18, 21, 26, 32, 38, and most recently (completed April 2019) 45 years, when 94.1% (*N* = 938) of the 997 participants still alive took part. A total of 875 (93% of age-45 participants) also completed MRI scanning. Scanned participants did not differ from other living participants on childhood social economic status (SES) or childhood intelligent quotient (IQ) (see attrition analysis in the Supplementary material). The relevant ethics committees approved each phase of the study, and informed consent was obtained from all participants.

### Measurement of cognitive ability

Cognitive ability in adulthood was assessed using the Wechsler Adult Intelligence Scale—IV (IQ score range, 40–160) at age 45 years (Weschler, 2008). Cognitive ability in childhood was assessed using the Wechsler Intelligence Scale for Children Revised (score range, 40–160) at ages 7, 9 and 11 with the mean for these three assessments used in analyses (Wechsler, 1974; Moffitt *et al.*, 1993). These tests are ideal for measuring child-to-adult cognitive decline because both tests are matched for content coverage and format, both were individually administered by trained psychometrists and both yield summary scores that are reliable at 95.

### Imaging parameters

Each participant was scanned using a Siemens Skyra 3 T scanner equipped with a 64-channel head/neck coil at the Pacific Radiology imaging centre in Dunedin, New Zealand. High-resolution structural images were obtained using a T_1_-weighted MP-RAGE sequence with the following parameters: Repetition Time (TR) = 2400 ms; Echo Time (TE) = 1.98 ms; 208 sagittal slices; flip angle = 9°; Field of View (FOV) = 224 mm; matrix = 256 × 256; slice thickness = 0.9 mm with no gap (voxel size 0.9 mm × 0.875 mm × 0.875 mm); and total scan time = 6 min and 52 s. 3D FLAIR images were obtained with the following parameters: TR = 8000 ms; TE = 399 ms; 160 sagittal slices; FOV = 240 mm; matrix = 232 × 256; slice thickness = 1.2 mm (voxel size 0.9 mm × 0.9 mm × 1.2 mm); and total scan time = 5 min and 38 s.

### Quantification of white matter hyperintensities

To identify and extract WMH volume, T_1_-weighted and FLAIR images for each participant were run through Unidentified Bright Object (UBO) Detector (Jiang *et al.*, 2018), a cluster-based, fully automated, pipeline that uses FMRIB’s Automated Segmentation Tool (Zhang *et al.*, 2001) to identify candidate clusters. Using K-nearest neighbours algorithms, clusters in the MRI images are classified as WMHs or non-WMHs (i.e. grey matter or cerebral spinal fluid) based on anatomical location, intensity and cluster size features. A Diffeomorphic Anatomical Registration through Exponentiated Lie template of 55 years or younger was used to best approximate the age of our cohort (Ashburner, 2007), and a grey matter mask was applied to decrease the chance of false positives. The resulting WMH probability maps were thresholded at 0.7, which is the suggested standard (Jiang *et al.*, 2018).

We chose the UBO pipeline because of its high reliability in our data (test–retest ICC = 0.87) and its out-of-sample performance (Jiang *et al.*, 2018). However, for additional quality assurance, every participant’s UBO-generated WMH map was visually inspected to check for false positives (e.g. areas such as the septum that appear similar to WMHs on FLAIR images). Of the 875 scanned participants who had at least one MRI scan, 867 had both a T_1_ image and an FLAIR image that are required to extract WMHs with UBO. A total of 843 participants were included in the final analysis after eight participants were removed for excessive UBO false positives, four participants were excluded because they had incidental findings that interfered with the UBO algorithm, three participants were removed for having multiple sclerosis and nine participants were excluded for missing IQ data in childhood or adulthood.

### Statistical analyses

All statistical analyses were done using R (v.3.4.5). First, descriptive statistics was generated for the sample as a whole (Table 1). Second, WMH volume was log-transformed for normality. Third, the associations between WMH volume (measured in cubic millimetres) and adult IQ and between volume and childhood IQ were tested using ordinary least squares multiple regression. Fourth, the association between volume and change in IQ was tested using ordinary least squares multiple regression. Measures of change in IQ were estimated by calculating residualized change scores. To do this, WMH volume was regressed on adult IQ, adjusting for childhood IQ. Sex and total brain volume were used as covariates in all analyses. All variables were *z*-transformed so that the resulting betas (*ß*) were standardized, allowing direct comparisons of effect sizes across regressions.

**Table 1:** Demographic characteristics for the 843 participants from the Dunedin Study included in the current analyses

	Total (*n* = 843)	Men (*n* = 428)	Women (*n* = 415)
Total brain volume (cm^3^)	1159.69 ± 117.12	1230.95 ± 96.06	1086.20 ± 87.84
BMI (kg/m^2^)	28.53 ± 5.81	28.54 ±4.73	28.53 ± 6.74
Glycated haemoglobin (mmol/mol)	38.65 ± 5.90	39.29 ± 6.37	37.98 ± 5.29
Cholesterol (mmol/l)	5.15 ± .99	5.32 ± 1.01	4.97 ± .93
Systolic blood pressure (mmHg)	121.32 ± 14.62	125.44 ± 13.89	117.06 ± 14.14
Diastolic blood pressure (mmHg)	80.31 ± 10.21	84.43 ± 9.41	76.04 ± 9.20
On blood pressure medication (*n*)	59	31	28
On cholesterol medication (*n*)	32	25	7
On medication for heart problems (*n*)	9	5	4
Heart attack (has/had) (*n*)	6	5	1
Atrial fibrillation (has/had) (*n*)	4	2	2
Cardiomyopathy (has/had) (*n*)	1	1	0
Blocked arteries (has/had) (*n*)	2	2	0
Current smokers (yes/no) (*n*)	176	95	81
Education attainment (%)			
% no qualification	14.6	17.8	11.3

Quantitative characteristics are reported as mean ± SD; qualitative characteristics are reported as number of participants (*n*) or percentage of participants (%). Units of measurement are denoted next to each variable. BMI = body mass index.

The premise and analysis plan for this project were pre-registered on https://sites.google.com/site/dunedineriskconceptpapers/documents. Analyses reported here were checked for reproducibility by an independent data-analyst, who recreated the code by working from the manuscript and applied it to a fresh dataset.

### Data availability

The dataset reported in the current article is not publicly available due to the lack of informed consent and ethical approval but is available from the corresponding author on reasonable request by qualified scientists. Requests require a concept paper describing the purpose of data access, ethical approval at the applicants’ university and provision for secure data access. Details are available at https://sites.google.com/site/dunedineriskconceptpapers/documents.

## Results

**Figure 1:**
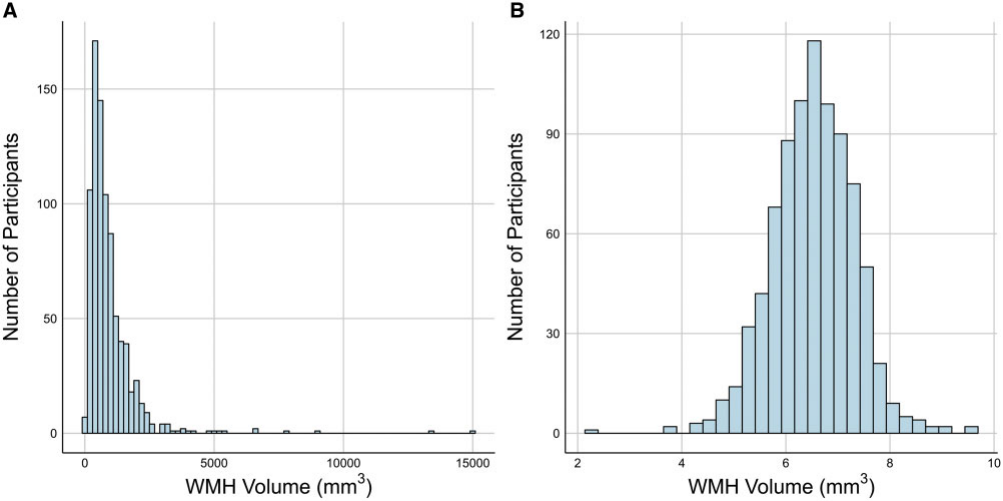
**Distribution of WMHs in 45-year-old participants from the Dunedin Study.** (**A**) Distribution of the raw WMH volumes. (**B**) Log-transformation of the volume distribution in **A**. All analyses reported used log-transformed volume.

**Figure 2:**
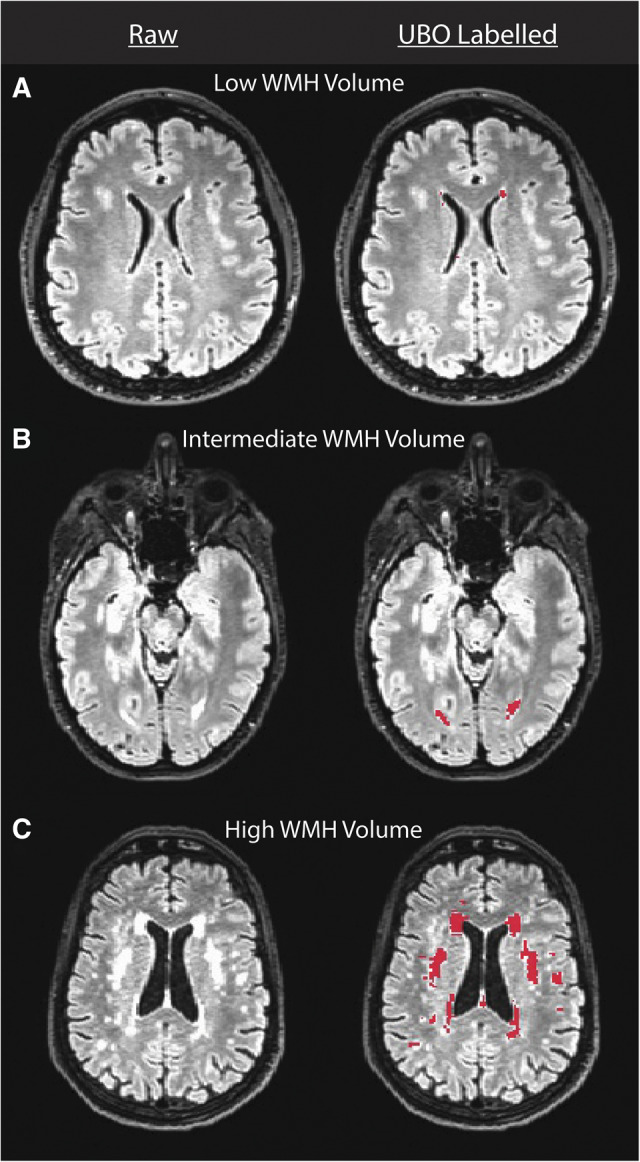
**Images depicting relatively low, intermediate and high WMH-load participants from the Dunedin Study.** The left column presents a raw FLAIR image for three representative participants with low, intermediate and high WMH load. The *right* column presents UBO labelling (red) of WMHs in the raw images from the *left* column. As can been seen in these images, WMHs were most common around the anterior and posterior horns of the lateral ventricles as expected. Note that UBO labelling in septal regions was removed from the estimation of WMH volume using an exclusion mask.

WMHs were common in the cohort, with an average volume of 953.50 mm^3^ (25th–75th quartile = 425.25–1,142.44 mm^3^, median = 681.75; Fig. 1). WMHs were most common around the anterior and posterior horns of the lateral ventricles (e.g. Fig. 2).

**Figure 3:**
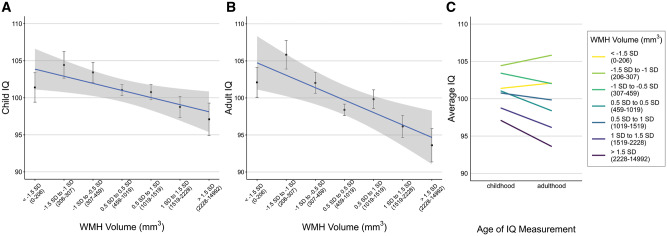
**Associations between WMH volume, cognitive ability and cognitive decline in 843 45-year-old participants from the Dunedin Study.** Study members in all panels are grouped into deciles defined by SDs from the mean WMH volume (mm^3^), ranging from −1.5 to 1.5 SDs in 0.5 SD increments. Sample sizes for each group from the lowest to the highest WMH volume were 57, 65, 128, 336, 133, 83 and 41. (**A**) Mean childhood IQ (average from measurements at ages 7, 9 and 11) for each of the WMH volume groups. (**B**) Mean adult IQ (measured at age 45) for the same groups. (**C**) Association between WMH volume and cognitive decline. The average IQ in childhood and adulthood in each of these is plotted to illustrate the increasing severity of cognitive decline as WMH volume increases. Cognitive decline is depicted here as the change from group mean childhood IQ score to group mean adult IQ score, for groups defined based on the level of white matter hyperintensities. All error bars display the standard error of the mean.

Larger WMH volume was associated with lower IQ in childhood (*ß* = −0.08, 95% CI = −0.15 to −0.02; *P* = 0.013; Fig. 3A); individuals with the highest volume (>1.5 SDs above the mean) had childhood IQs that were 4.80 points lower on average than individuals with the lowest volume (<1.5 SDs below the mean). This difference was exacerbated in adulthood; larger WMH volume was associated with lower IQ (*ß* = −0.15, 95% CI = −0.22 to −0.09; *P* < 0.001; Fig. 3B) and individuals with the highest volume had adult IQs that were 8.91 points lower than those with the lowest volume. Lastly, participants with larger WMH volume experienced more cognitive decline by midlife (*ß* = −0.09, 95% CI = −0.13 to −0.02; *P* < 0.001; Fig. 3C). A sensitivity analysis using a difference score (adult IQ − childhood IQ) as a measure of change in IQ rather than a residualized change score did not change the results. Additional sensitivity analyses controlling for the possible confounding effects of mean arterial pressure at age 45 and childhood SES on the association between WMH volume and cognitive decline showed only minor attenuation in effect sizes, and all but one association remained significant (*P* < 0.05; Supplementary Table 1). Specifically, the addition of childhood SES as a covariate attenuated the relationship between childhood IQ and WMH volume because SES and IQ are known to be closely related during childhood.

Secondary analyses using the four sub-indexes of adult IQ showed that larger WMH volume was associated with lower scores on processing speed (*ß* = −0.14, 95% CI = −0.20 to −0.07; *P* < 0.001), verbal comprehension (*ß* = −0.14, 95% CI = −0.21 to −0.07; *P* < 0.001) and perceptual reasoning (*ß* = −0.13, 95% CI = −0.19 to −0.06; *P* < 0.001). There was also a trending association between larger volume and decreased working memory ability (*ß* = −0.06, 95% CI = −0.13 to 0.00; *P* = 0.06).

## Discussion

In a population-representative birth cohort of individuals now in midlife, we found that WMHs are (i) common, (ii) associated with cognitive abilities in childhood and midlife and (iii) associated with cognitive decline from childhood to midlife. While there is robust evidence that WMHs are related to cognitive decline in older adults (Debette and Markus, 2010; Prins and Scheltens, 2015), our study provides initial evidence that this relationship begins by midlife.

These findings demonstrate that the link between WMHs and early signs of cognitive decline is detectable decades before clinical symptoms of ADRD typically emerge. Longitudinal studies have shown that WMHs tend to grow and expand from existing lesions and that higher baseline volumes predict faster accumulation of WMHs and more rapid cognitive decline in older adults (Maillard *et al.*, 2012; Prins and Scheltens, 2015). Thus, our findings suggest that WMHs may be a surrogate biomarker for identifying individuals in midlife who are at risk for future clinically significant cognitive decline or ADRD. Our results further show that accumulation of WMHs in midlife already indicates mild cognitive decline. This is important because even sub-clinical cognitive decline impacts daily functioning and psychological well-being (Tucker-Drob, 2011).

Interestingly, our results also showed a modest association between low childhood IQ and WMHs in midlife. This finding suggests at least two potential pathways for the development of WMHs. The first possibility is that children with lower IQs tend to be born into or seek out environments that lead to higher rates of neurodegeneration (e.g. poor nutrition, smoking, drug abuse, lead exposure). Over time these exposures may lead to negative health outcomes, such as higher risk for cardiovascular disease, brain damage and higher blood pressure, which contribute to increased WMH volume in midlife. This perspective would suggest that interventions to limit neurodegenerative environmental exposures (e.g. anti-drug messaging, better nutrition) in high-risk children could limit the burden of cognitive decline and ADRD later in life. The second possibility is that low IQ is an indicator of lower overall brain integrity that was present early in life (Deary, 2012). This perspective suggests that the association between low childhood IQ and midlife WMH is driven by a higher vulnerability to tissue damage and faster neurodegeneration in low-IQ children, given the same lifetime exposures. This further suggests a need for interventions that increase brain resiliency and boost tissue regeneration in those at highest risk (e.g. cognitive training or pharmaceutical intervention). A limitation of our study is the lack of childhood neuroimaging to assess the development of WMHs across the lifespan, although it should be noted that no sample with WMH measures in midlife would have childhood WMH measures, because cohorts of non-patient children did not have MRI imaging 40 years ago. As such, our findings point to the need to investigate these possible mechanistic pathways in future studies with child-to-adult imaging data.

Intervention efforts targeting WMHs as a surrogate biomarker in older adults have had mixed results (Prins and Scheltens, 2015). One reason for this inconsistency could be that older adults have accumulated decades of irrevocable age-related tissue damage. Given that prevention of damage is often more efficacious than reversal of damage (Sperling *et al.*, 2014; Moffitt *et al.*, 2017), particularly in the brain, our results suggest that lifestyle and pharmaceutical interventions aimed at slowing the progression of WMHs in midlife may be promising complements to interventions in older adults. Due to their compounding growth during aging, WMHs may be especially useful for selecting individuals in midlife who are at the highest risk for future cognitive decline and who may most benefit from early prevention.

## Supplementary Material

fcz041_Supplementary_Data

## Abbreviations

ADRD: Alzheimer’s disease and related dementias
FLAIR: fluid-attenuated inversion recovery
MRI: magnetic resonance imaging
WMH: white matter hyperintensity
