# Supplementary material for: White matter hyperintensities are common in midlife and already associated with cognitive decline
Source: Brain Commun. 2019 Dec 9;1(1):fcz041. doi: 10.1093/braincomms/fcz041 (PMC6928390; doi:10.1093/braincomms/fcz041)
Supplement: fcz041_Supplementary_Data [file fcz041_supplementary_data.pdf]

Supplementary Material

Phase 45 Attrition Analysis

We conducted an attrition analysis using childhood intelligence quotient (IQ; Supplementary Fig.1) and socioeconomic status (SES; Supplementary Fig. 2) to determine whether participants in the Phase 45 data collection were representative of the original cohort.

Attrition Analysis of Childhood IQ in Phase 45

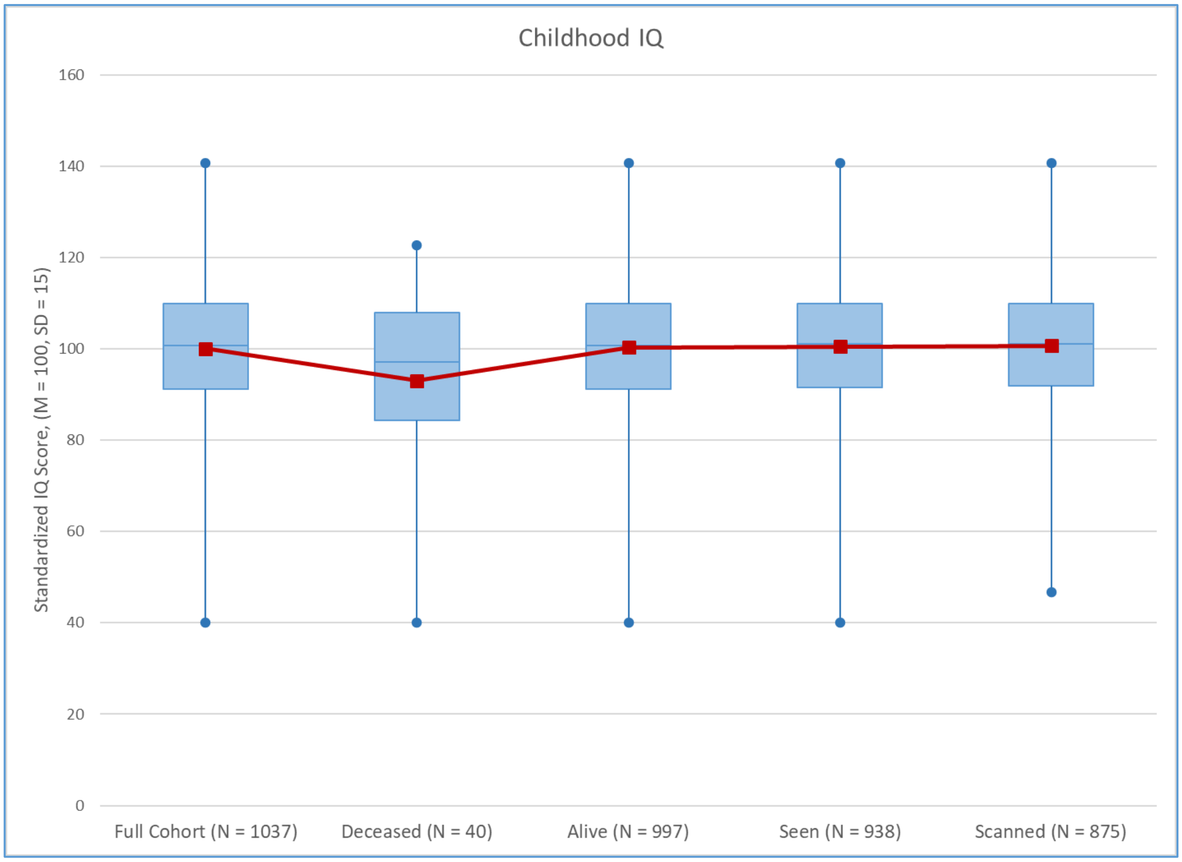

Supplementary Figure 1. No significant differences in childhood IQ were found between the full cohort, those still alive, those seen at Phase 45 or those scanned at Phase 45. Those who were deceased by the Phase 45 data collection had significantly lower childhood IQ's than those who were still alive ($t = 2.09$, $p = 0.04$).

Attrition Analysis of Childhood SES in Phase 45

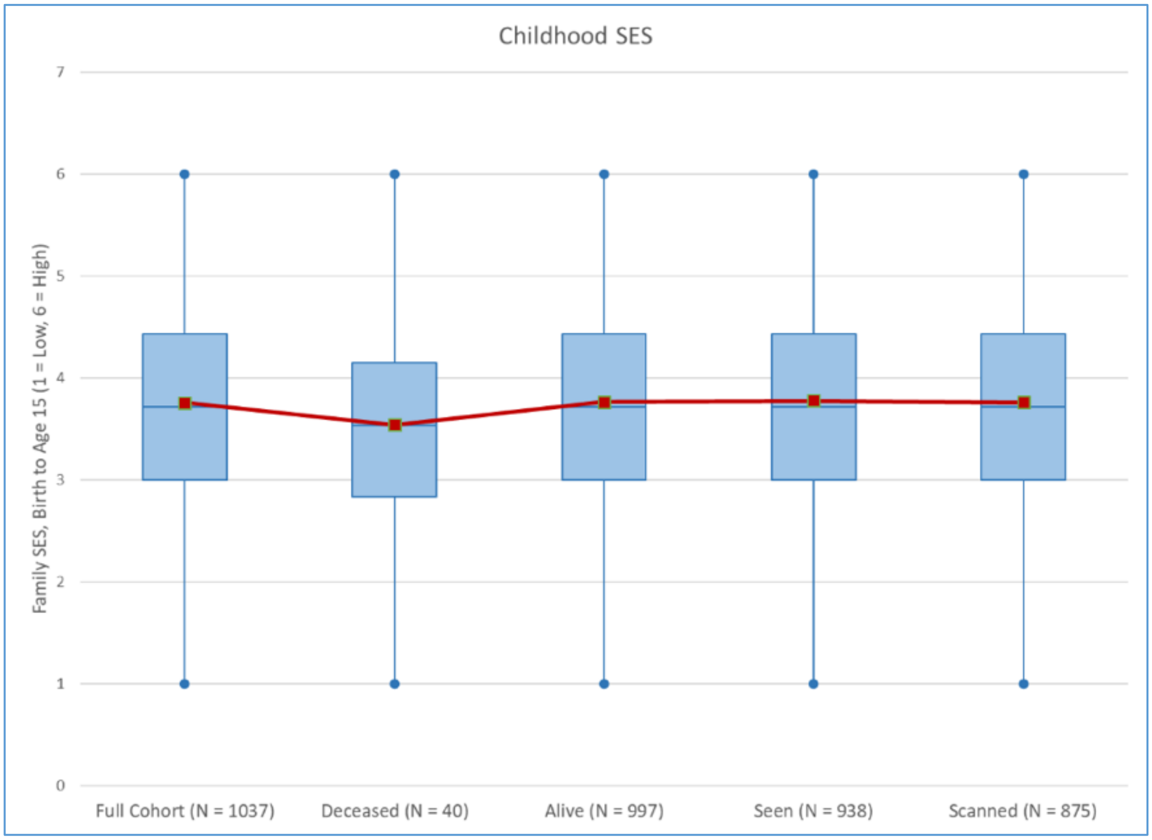

Supplementary Figure 2. No significant differences were found between the full cohort, those deceased, those alive, those seen at Phase 45 or those scanned at Phase 45 on childhood SES.

Sensitivity Analyses	Standardized β (95% CIs)	P value
**Association between Adult Cognitive Function (age 45) and:**		
WMH	-0.15 (-0.22 to -.09)	<.001
WMH (adjusting for mean arterial blood Pressure)	-0.13 (-0.21 to -0.08)	<.001
WMH (adjusting for childhood SES)	-0.15 (-0.19 to -0.06)	<.001
**Association between Childhood Cognitive Function and:**		
WMH	-0.08 (-0.15 to -0.01)	0.017
WMH (adjusting for mean arterial blood Pressure)	-0.07 (-0.14 to -0.01)	0.034
WMH (adjusting for childhood SES)	-0.05 (-0.11 to 0.01)	0.125
**Association between Cognitive Decline (Childhood to Age 45) and:**		
WMH	-0.09 (-0.13 to -0.05)	<.001
WMH (adjusting for mean arterial blood Pressure)	-0.09 (-0.13 to -0.05)	<.001
WMH (adjusting for childhood SES)	-0.09 (-0.13 to -0.05)	<.001

Supplemental Table 1. Sensitivity analyses demonstrating the incremental validity of WMH to predict cognitive function and cognitive decline over and above arterial blood pressure and childhood socioeconomic status (SES).
